# Revisiting the Lamotrigine-Mediated Effect on Hippocampal GABAergic Transmission

**DOI:** 10.3390/ijms17071191

**Published:** 2016-07-22

**Authors:** Yu-Yin Huang, Yu-Chao Liu, Cheng-Ta Lee, Yen-Chu Lin, Mong-Lien Wang, Yi-Ping Yang, Kaung-Yi Chang, Shih-Hwa Chiou

**Affiliations:** 1 Institute of Clinical Medicine, National Yang-Ming University, Taipei 112, Taiwan; huang_yuyin@yahoo.com.tw (Y.-Y.H.); molly0103@gmail.com (Y.-P.Y.); 2 Department of Anesthesiology, Cheng Hsin General Hospital, Taipei 112, Taiwan; rainnyhunter@gmail.com; 3 Institute of Neuroscience and Brain Research Center, National Yang-Ming University, Taipei 112, Taiwan; tsohchi@hotmail.com (C.-T.L.); g39601013@gmail.com (Y.-C.L.); 4 Institute of Pharmacology, National Yang-Ming University, Taipei 112, Taiwan; monglien@gmail.com; 5 Department of Medical Research, Taipei Veterans General Hospital, Taipei 112, Taiwan; 6 Department of Neurological Surgery, Tri-Service General Hospital, National Defense Medical Center, Taipei 112, Taiwan; 7 Department of Anesthesiology, Taipei Veterans General Hospital, Taipei 112, Taiwan; kychang@vghtpe.gov.tw

**Keywords:** Lamotrigine, GABAergic interneuron, inhibitory postsynaptic current, voltage-gated sodium channel, hyperpolarization-activated current

## Abstract

Lamotrigine (LTG) is generally considered as a voltage-gated sodium (Na_v_) channel blocker. However, recent studies suggest that LTG can also serve as a hyperpolarization-activated cyclic nucleotide-gated (HCN) channel enhancer and can increase the excitability of GABAergic interneurons (INs). Perisomatic inhibitory INs, predominantly fast-spiking basket cells (BCs), powerfully inhibit granule cells (GCs) in the hippocampal dentate gyrus. Notably, BCs express abundant Na_v_ channels and HCN channels, both of which are able to support sustained action potential generation. Using whole-cell recording in rat hippocampal slices, we investigated the net LTG effect on BC output. We showed that bath application of LTG significantly decreased the amplitude of evoked compound inhibitory postsynaptic currents (IPSCs) in GCs. In contrast, simultaneous paired recordings from BCs to GCs showed that LTG had no effect on both the amplitude and the paired-pulse ratio of the unitary IPSCs, suggesting that LTG did not affect GABA release, though it suppressed cell excitability. In line with this, LTG decreased spontaneous IPSC (sIPSC) frequency, but not miniature IPSC frequency. When re-examining the LTG effect on GABAergic transmission in the cornus ammonis region 1 (CA1) area, we found that LTG markedly inhibits both the excitability of dendrite-targeting INs in the stratum oriens and the concurrent sIPSCs recorded on their targeting pyramidal cells (PCs) without significant hyperpolarization-activated current (I_h_) enhancement. In summary, LTG has no effect on augmenting I_h_ in GABAergic INs and does not promote GABAergic inhibitory output. The antiepileptic effect of LTG is likely through Na_v_ channel inhibition and the suppression of global neuronal network activity.

## 1. Introduction

Lamotrigine (LTG), a broad-spectrum antiepileptic drug (AED), is effective and well tolerated in the treatment of partial and generalized seizures among adults, women in pregnancy and children [1,2,3]. In addition, LTG is also used for other non-epileptic conditions, like bipolar disorder [4,5,6]. Although it acts on multiple molecular targets in the central nervous system, LTG mainly serves as a voltage-gated Na^+^ (Na_v_) channel blocker [7,8,9,10]; thus, it can suppress the neuronal excitability and consequently block neurotransmitter release. However, recent studies from brain slice recordings show that LTG increases the frequency of both spontaneous inhibitory postsynaptic currents (sIPSCs) and miniature IPSCs (mIPSCs) in the entorhinal cortex [11,12]. Furthermore, LTG also facilitates GABAergic transmission onto hippocampal cornus ammonis region 1 (CA1) pyramidal cells (PCs) [13]. The enhanced GABAergic transmission correlates with the increased spontaneous firing rate of a specific population of CA1 interneurons (INs) possibly via potentiation of hyperpolarization-activated cation channels (I_h_ or HCN channels) by LTG [13,14]. Notably, I_h_ channels are highly expressed in the majority of the dendrite-targeting CA1 INs [15,16,17].

In addition to dendrite-targeting INs, principal neurons are innervated by soma-targeting INs including fast-spiking basket cells (BCs). In the dentate gyrus (DG), BCs are thought to contribute to the majority of GABAergic transmission onto principal neurons, i.e., granule cells (GCs) [18]. A recent study shows that fast-spiking BCs in the DG also express I_h_ channels, and the blockade of I_h_ channels by the specific antagonist 4-ethylphenylamino-1,2-dimethyl-6-methylaminopyrimidinium chloride (ZD7288) decreases mIPSCs in GCs [19]. Furthermore, in the cerebellum, I_h_ current is recorded from axonal terminals of BCs. Similarly, inhibition of I_h_ by ZD7288 reduces the frequency and amplitude of sIPSCs recorded in Purkinje cells, one of the principal synaptic targets of BCs [20]. Hippocampal BCs are known to generate high-frequency action potentials (APs) in vivo and in vitro and are thus susceptible to the Na_v_ channel blocking effect of LTG. On the other hand, if LTG acts as an I_h_ channel enhancer [13,14], it can depolarize BCs and support persistent discharges [21], thereby promoting GABA release. Hence, the net effect of LTG on BC output still remains a paradoxical question.

In this study, by using patch clamp recordings in a rat hippocampal slice, we first investigated the effect of LTG on GABAergic transmission in the DG, as well as in the CA1 area. Second, we further evaluated the LTG effect on the active and passive membrane properties of presynaptic INs, including soma-targeting or dendrite-targeting INs. Finally, we confirmed the existence of the LTG effect on I_h_ channel-mediated currents. Our findings showed that LTG greatly decreased GABAergic inhibition through the suppression of IN excitability in both the GCs and CA1 PCs, respectively, without affecting I_h_ currents.

## 2. Results

### 2.1. Bath Application of LTG Resulted in the Reduction of Compound IPSCs

**Figure 1:**
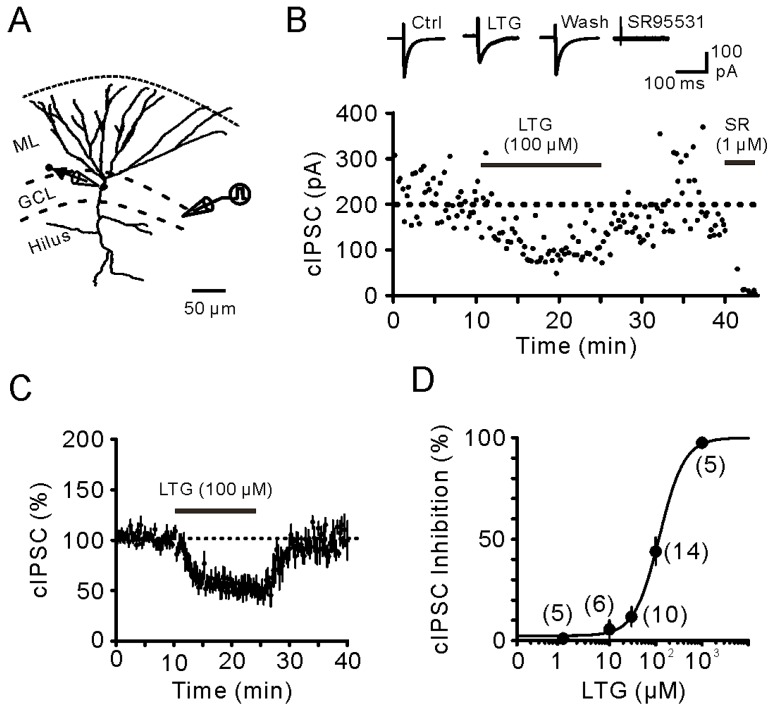
Suppression of somatic GABAergic transmission onto GCs by LTG. (**A**) Schematic of experiment configuration: A stimulating electrode placed at a distance of 100–200 µm from a recorded GC within the GCL. ML, molecular layer; GCL, granule cell layer; (**B**) (**Top**) Exemplar average compound IPSCs (cIPSCs) (15–20 sweeps) recorded in the control, in LTG (100 µM), after LTG washout and after the addition of Gabazine (SR95531); (**Bottom**) Plot of the peak amplitudes of cIPSC against time; (**C**) Plot of the mean peak amplitude of cIPSC (*n* = 10) against time. Data were normalized to the baseline before LTG application. Symbols indicate the mean; error bars indicate SEM; (**D**) Dose-response relationship of cIPSC inhibition by LTG (1, 10, 30, 100 and 1000 µM). Data fitted to a single Hill equation with IC_50_ = 121.2 µM and Hill coefficient = 1.51. Each point represents the average from 5–14 experiments, as given in parentheses; error bars indicate SEM.

Fast-spiking BCs innervate the perisomatic domain of dentate GCs and mediate stable and powerful inhibition [22,23,24]. Recent studies showed that I_h_ channels in fast-spiking BCs critically regulate their membrane potential and persistent firing properties [19,21]. LTG, which is considered as an I_h_ enhancer, is shown to facilitate spontaneous IPSCs in CA1 PCs [13,14]. We thus tested whether LTG increased GABAergic transmission at BC–GC synapses in the DG. Pharmacologically-isolated compound IPSCs (cIPSCs) were recorded from GCs by local stimulation of the putative BC axons in the GC layer (GCL) in the presence of kynurenic acid (KA, 2 mM), the antagonist for ionotropic glutamate receptors (Figure 1A). In the voltage clamp at −70 mV, the recorded cIPSCs were inward because of the high concentration of Cl¯ (144 mM) in the pipette solution. As illustrated in Figure 1B, bath application of LTG (100 µM) markedly and reversibly inhibited cIPSCs, which were further abolished by the application of the GABA_A_ receptor antagonist Gabazine (SR95531) (1 µM) (Figure 1B). Quantification of the cIPSCs reduction showed that LTG (100 µM) reduced the mean amplitudes to 51.6% ± 4.4% of control (*n* = 14, *p <* 0.01, Figure 1C). To evaluate the dose-dependent effect of LTG-mediated cIPSCs’ reduction, we recorded cIPSCs at different LTG concentrations. As shown in Figure 1D, the reduction in the mean cIPSC amplitude was dependent on LTG concentrations. The dose-response curve was fitted with the Hill equation, which showed that the half maximal inhibitory concentration (IC_50_) was 121.2 µM, and the Hill coefficient was 1.51 (Figure 1D).

### 2.2. LTG Had No Effects on Unitary IPSCs

**Figure 2:**
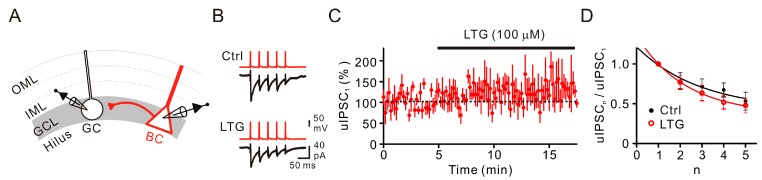
LTG had little effect on GABA release at BC-GC synapses. (**A**) Schematic diagram showing the BC-GC paired recording configuration. OML, outer molecular layer; IML, inner moleucular layer; (**B**) The 25-Hz bursts of five presynaptic APs (**red**) and postsynaptic unitary IPSC (uIPSC) traces (**black**, average of 25–30 sweeps) in the control (Ctrl) and after bath perfusion of LTG (100 µM); (**C**) Summary of the normalized uIPSC_1_ mean peak amplitude from five BC-GC pairs against time. Symbols indicate the mean; error bars indicate SEM; (**D**) Mean ratio of uIPSC_n_/uIPSC_1_ plotted against the number within the train (*n*).

The reduction of compound IPSCs can be explained by several potential mechanisms, including reduction on the release probability and neuronal excitability [19,22]. To examine the direct effect of LTG on GABA release, we made paired recordings between synaptically-coupled BCs and GCs (Figure 2A). Unitary postsynaptic IPSCs recorded in GCs were evoked by applying brief current pulses to the presynaptic BCs. A 25 Hz burst of five APs was repetitively applied every 10 s (Figure 2B). When the peak amplitude of the first unitary IPSC (uIPSC_1_) was plotted against time, the mean magnitude remained unaltered after the LTG (100 µM) application (128% ± 27% of control, *n* = 6 pairs, *p* = 0.44, Figure 2C). Furthermore, there were no changes in the multiple pulse ratio (uIPSC_5_/uIPSC_1_) (control, 0.53 ± 0.11; LTG, 0.48 ± 0.09, *p =* 0.13, Figure 2D). Overall, these results indicated that LTG had no significant effects on the release probability of GABAergic transmission at the BC-GC synapse. The inhibitory effect of LTG on cIPSCs was likely attributed to the reduction of neuronal excitability.

### 2.3. LTG Decreased sIPSCs, but Not mIPSCs

**Figure 3:**
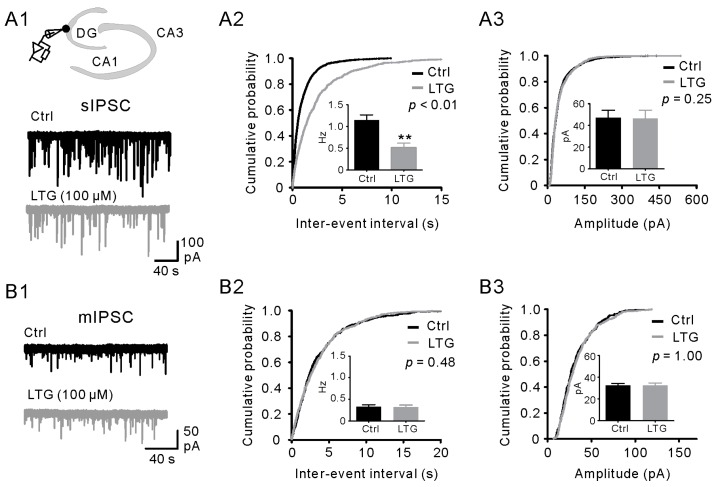
LTG decreased the frequency of spontaneous IPSCs (sIPSCs), but not miniature IPSCs (mIPSCs) in GCs. (**A1**) (**Top**) Recording configuration: Whole-cell voltage-clamp recordings from a GC at −70 mV in the DG. CA1, cornus ammonis region 1; (**Middle** and **bottom**) Traces of sIPSCs recorded before (**black**) and after (**gray**) LTG (100 µM) application; (**A2**,**A3**) Cumulative distributions of sIPSC inter-event intervals (**A2**) and amplitudes (**A3**) from the control (**black**; *n* = 10) and LTG (**gray**; *n* = 10). Insets show the bar graph summaries of the averages. ** *p <* 0.01; (**B1**) (**Top**) Traces of mIPSCs recorded at −70 mV in the control (**black**); (**Bottom**) Events in the presence of LTG (100 µM) application (**gray**); (**B2**,**B3**) Cumulative distributions of mIPSC inter-event intervals (**B2**) and amplitude (**B3**) in the control (**black**, *n* = 10) and LTG (**gray**, *n* = 10). Insets show the bar graph summaries of the averages.

Spontaneous IPSCs (sIPSCs) comprise action potential (AP)-dependent and AP-independent GABA release. LTG is a well-known blocker for voltage-gated sodium channels. Since we found that LTG has no effects on GABA release, we hypothesized that LTG can decrease sIPSC frequency without affecting mIPSCs. To test this hypothesis, we first recorded sIPSCs from GCs, the main target cells of BCs, before and after LTG application (Figure 3A1). Consistent with the results of cIPSC experiments (Figure 1), 100 µM of LTG significantly reduced the frequency of sIPSCs (control, 1.2 ± 0.1 Hz; LTG, 0.52 ± 0.09 Hz, *n* = 10, *p* < 0.01, Figure 3A2), whereas the mean amplitude was unchanged (control, 47.06 ± 6.89 pA; LTG, 46.4 ± 7.7 pA, *p* = 0.25, Figure 3A3). We next recorded the mIPSCs from GCs in the presence of Tetrodotoxin (TTX) (1 µM). As illustrated in Figure 3B1, LTG had no effect on both mIPSC frequency (control, 0.32 ± 0.05 Hz; LTG, 0.31 ± 0.05 Hz, *n* = 10, *p* = 0.48, Figure 3B2) and mIPSC amplitude (control, 32.6 ± 1.9 pA; LTG, 32.6 ± 2.3 pA, *p* = 1.00, Figure 3B3). Taken together, our results suggested that LTG-induced depression of GABAergic transmission may be mediated by the suppression of presynaptic excitability.

### 2.4. LTG Inhibited the Axonal and Cellular Excitability of BCs in the DG

**Figure 4:**
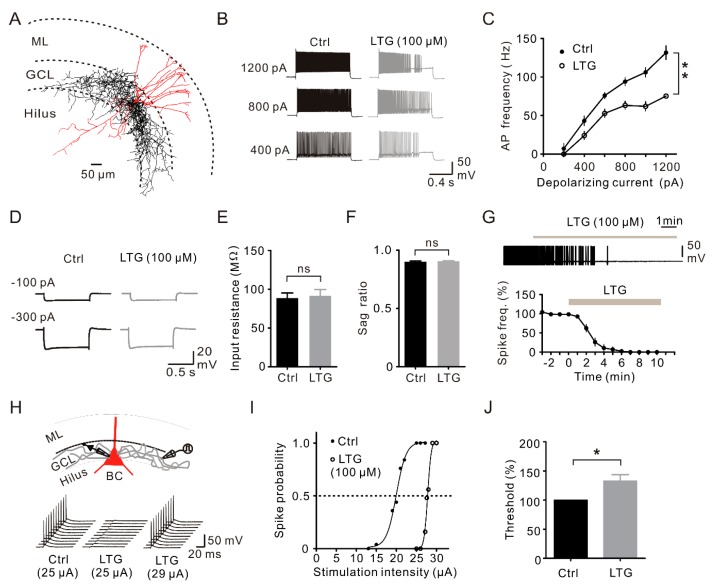
LTG suppressed BC excitability. (**A**) Reconstruction of a biocytin-filled BC whose axon arborized in the GCL. Soma and dendrites are shown in red, and axons are in black; (**B**) Exemplar traces of APs evoked by 1-s depolarizing current pulses (400, 800, 1200 pA) in the presence of synaptic blockers. Black traces, control; gray traces, LTG. Cells were held at −70 mV by holding the current adjustment throughout the experiment; (**C**) Mean AP frequency plotted against the injected current (*n* = 10). ** *p <* 0.01; (**D**) Exemplar voltage traces recorded during 1-s hyperpolarizing current pulses (−100 pA and −300 pA, respectively) under whole-cell current-clamp before (**black** traces) and after 100 µM LTG (**gray** traces) application; (**E**,**F**) Summary bar graphs of the effect of 100 µM LTG on (**E**) input resistance and (**F**) the sag ratio (voltage change at the end of the 1-s pulse/maximal voltage change or −300 pA current injection) with no significant difference under control and LTG conditions (*n* = 9). ns, no significance; (**G**) Summary of the plot of the spontaneous spike frequency against time illustrating the effect of 100 µM LTG (*n* = 10). BCs were slightly depolarized to fire persistent APs by sustained somatic current injection (100–200 pA); (**H**) (**Top**) A stimulating electrode (monopolar glass pipette) placed in the GCL at a distance of 100–300 µm from the recorded BC; (**Bottom**) 10 consecutive spikes of APs in the control (stimulus intensity 25 µA) (**left**), in the presence of 100 µM LTG at the same stimulus intensity (**middle**) and in the presence of 100 µM LTG after the increase in stimulation intensity (**right**, 29 µA); (**I**) Spike probability plotted against stimulus intensity in the control (**black**) and after LTG (**gray**). The dashed line indicates that LTG increases the threshold for spike initiation (current leading to 50% successes); (**J**) Summary of the LTG effect on the AP threshold. Data were normalized to the control from five BCs. * *p* < 0.05.

The BCs represent a major type of hippocampal INs [19] and largely contribute to IPSCs received by GCs [18]. To examine whether LTG affected BC excitability, we evoked APs in BCs in the presence of synaptic blockers by injecting depolarizing current pulses in current-clamp recordings. Putative BCs were selected by their larger soma in the GC layer near the hilar border (Figure 4A), fast spiking AP phenotype (≥70 Hz) and little sag with input resistance <170 MΩ [19,25,26]. Following bath application of LTG, BCs were less excitable compared to the control condition (Figure 4B), which was illustrated by reduced AP firing frequency across the range of current steps (*n* = 10, *p* < 0.01, Figure 4C). In addition, there were no significant differences in input resistance (control, 88.61 ± 6.62 MΩ; LTG, 91.5 ± 8.09 MΩ, *n* = 10, *p* = 0.79, Figure 4D,E) and the sag ratio (control, 0.90 ± 0.02; LTG, 0.91 ± 0.02; *p* = 1.0, Figure 4D,F) before and after LTG application. On the other hand, the recorded BCs had resting membrane potentials between −60 and −70 mV and did not generate spontaneous spikes. We further injected a 100~200 pA current to depolarize BCs and induced spontaneous firing. LTG subsequently blocked the firing activity in 10 minutes (Figure 4G).

To further test the LTG effect on BC axonal excitability, BC axons were stimulated extracellularly, and antidromic APs were recorded at the soma under current-clamp configurations (Figure 4H, top). The suprathreshold stimuli initiated APs with 100% reliability in the control condition. Notably, the reliability of AP generation diminished after the application of LTG. To quantify the shift in stimulation threshold, the spike probability was plotted against stimulus intensity (Figure 4I). On average, LTG increased the threshold of stimulation intensity (defined as the intensity reaching 50% success) to 132% ± 8% of the control (*n* = 5, *p* < 0.05, Figure 4J). These findings indicated that LTG reduced axonal excitability of fast-spiking BCs, which underlies the suppression of IPSCs in GCs.

### 2.5. LTG Suppressed sIPSCs Rather than mIPSCs in CA1 PCs

**Figure 5:**
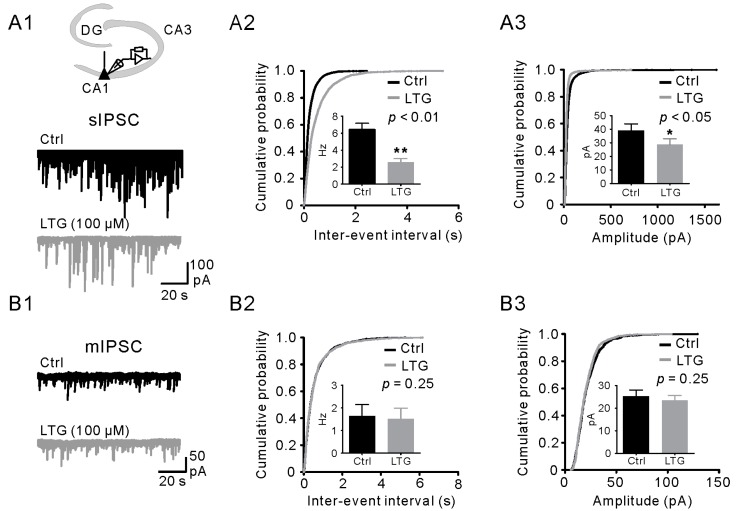
LTG decreased sIPSC frequency in CA1 PCs without affecting mIPSC frequency. (**A1**) (**Top**) Whole-cell voltage-clamp recordings from a CA1 PC; (**Middle** and **bottom**) Traces of sIPSCs recorded at −70 mV in the control (**black**) and LTG (100 µM) (**gray**); (**A2**,**A3**) Cumulative distributions of sIPSC inter-event intervals (**A2**) and amplitudes (**A3**) from the control (**black**) and LTG (**gray**). Insets show the bar graph summaries of the cell averages (*n* = 8) for the frequency (**A2**) and amplitude (**A3**). ** *p* < 0.01; (**B1**) (**Top**) Traces of mIPSCs recorded at −70 mV in control conditions (**black**); (**Bottom**) Events in the presence of LTG (100 µM) application (**gray**); (**B2**,**B3**) Cumulative distributions of mIPSC inter-event intervals (**B2**) and amplitudes (**B3**) from the control (**black**) and LTG (**gray**). Insets show the bar graph summaries of the ell averages (*n* = 6) for the frequency (**B2**) and amplitude (**B3**), respectively. * *p* < 0.05.

A previous study reported that LTG activates the I_h_ channel in stratum oriens (SO) INs and excites the presynaptic INs, thereby leading to increased frequency of sIPSCs in PCs [13]. Here, in our model, we examined the effects of LTG on sIPSCs in CA1 PCs (Figure 5A1). Consistent with our findings in the DG, the frequency of sIPSCs was greatly reduced from 6.43 ± 0.73 Hz down to 2.58 ± 0.42 Hz (*n* = 8, *p* < 0.01, Figure 5A2), whereas the mean amplitude was changed from 39.02 ± 4.94 pA down to 28.80 ± 4.18 pA (*n* = 8, *p* < 0.05, Figure 5A3). In contrast to sIPSCs (Figure 5B1), LTG has no significant effects on either mIPSC frequency (frequency in control, 1.95 ± 0.51 Hz; LTG, 1.81 ± 0.48 Hz, *n* = 6, *p* = 0.25, Figure 5B2) or amplitude (control, 24.94 ± 2.49 pA; LTG, 23.53 ± 2.13 pA, *n* = 6, *p =* 0.25, Figure 5B3). These data indicated that LTG also downregulates GABA transmission in the CA1, which is caused by the suppression of presynaptic excitability.

### 2.6. LTG Inhibited the Excitability of CA1 O-LM INs without Affecting the I_h_

Reductions of sIPSCs, but not mIPSCs in CA1 PCs suggested that LTG mainly reduces neuronal activity. Besides soma-targeting Ins, such as BCs, dendrite-targeting INs, including oriens-lacunosum moleculare (O-LM) INs in CA1 stratum oriens (SO), provide inhibition of PCs. O-LM INs exhibit a prominent sag response to hyperpolarizing current pulse injection [15,27,28,29].

**Figure 6:**
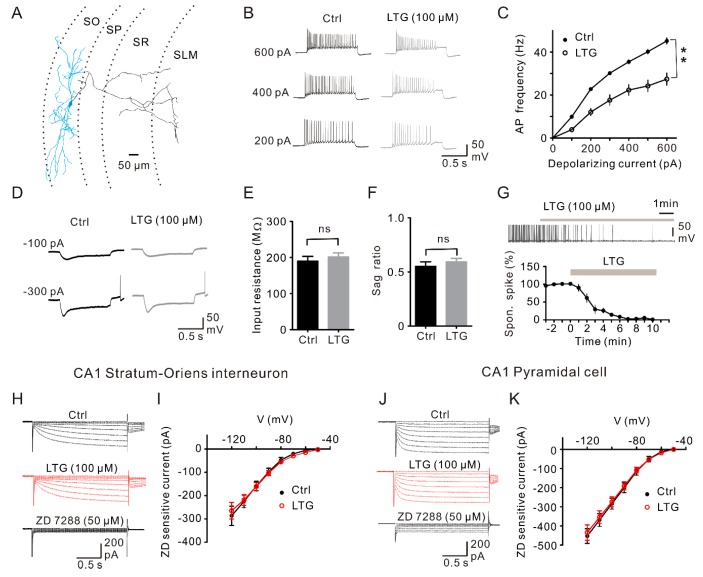
LTG suppressed oriens-lacunosum moleculare (O-LM) IN excitability without affecting I_h_. (**A**) Reconstruction of a biocytin-filled O-LM IN. Soma and dendrite are shown in light blue, and axons are in black. SLM, stratum lacunosum moleculare; SR, stratum radiatum; SP, stratum pyramidal; SO, stratum oriens; (**B**) Exemplar AP traces from whole-cell current-clamp recordings of control (**black**) and LTG (**gray**) in an O-LM IN evoked by 1-s depolarizing current steps in the presence of synaptic blockers; (**C**) The AP frequency was plotted against the stepwise stimulus intensity (100~600 pA) and revealed significant inhibition by LTG (*n* = 8). ** *p <* 0.01; (**D**) Voltage responses to 1-s hyperpolarizing (−100 pA and −300 pA) current pulses from the same cell were recorded; (**E**) Summary of the input resistance. ns, no significance; (**F**) Summary of the sag ratio (voltage change at the end of the 1-s pulse/maximal voltage change or −300 pA current injection) with no significant difference under the control and LTG condition; (**G**) (**Top**) In a resting membrane potential around −60 mV, an O-LM IN generated spontaneous firing and was abolished by LTG (100 µM); (**Bottom**) Plot of normalized induced spontaneous spike frequency against time illustrating the effect of 100 µM LTG (*n* = 8); (**H**) Currents activated by hyperpolarizing pulse from a holding potential of −50 mV−120 mV with an increment of 10 mV under voltage clamp mode from a representative O-LM IN in the control condition, with LTG (100 µM) and 4-ethylphenylamino-1,2-dimethyl-6-methylaminopyrimidiniumchloride (ZD7288) (50 µM); (**I**) Plotting the ZD7288-sensitive current (current of the control or LTG with digital subtraction of the ZD current) against hyperpolarizing voltage steps before and after LTG application demonstrated that LTG had no significant effect on I_h_ (*n* = 6); (**J**) The same protocol as (H) was made in CA1 PC; (**K**) Plotting the ZD7288-sensitive current against hyperpolarizing voltage steps before and after LTG application demonstrated that LTG had no significant effect on I_h_ (*n* = 6).

Previous studies showed that LTG increases the frequency of APs and changes the input resistance, as well as the sag response [13,28]. To test the LTG effects on the membrane excitability of O-LM INs (Figure 6A), we evoked O-LM IN firing by delivering 1-s depolarizing current pulses. In contrast to previous studies, LTG at the same concentration as previous reports (100 µM) significantly suppressed the firing frequency (*n* = 8, *p* < 0.01, Figure 6B,C). Meanwhile, the spontaneous firing of O-LM INs was abolished by LTG (Figure 6D). Moreover, there were no significant differences on input resistance (Ctrl, 190.2 ± 13.02 MΩ; LTG, 201.7 ± 11.15 MΩ, *n* = 7, *p* = 0.29, Figure 6D,E) and the sag ratio change (voltage change at the end of the 1-s pulse/maximal voltage change for a −300-pA current injection) [27] before and after LTG application (Ctrl, 0.55 ± 0.04; LTG, 0.59 ± 0.03, *n* = 7, *p* = 0.43, Figure 6D,F).

Finally, we determined whether LTG affected I_h_ in CA1 O-LM INs. To evoke the I_h_ current, we made voltage-clamp recordings from O-LM INs at −50 mV and delivered 2-s hyperpolarizing voltage pulses (10-mV increments from −50 down to −120 mV; Figure 6H). Consistent with the non-changed input resistance, slowly non-inactivating inward currents were also not significantly affected by LTG (*n* = 6, *p* = 0.918, Figure 6I). The same experiment was also performed in CA1 PCs, which abundantly express the I_h_ channel [14,30]. The results showed that LTG had no remarkable effects on the I_h_ in CA1 PCs (*n* = 6; *p* = 0.948, Figure 6J,K).

## 3. Discussion

Na_v_ and HCN channels are highly expressed in certain types of soma- and dendrite-targeting GABAergic INs, such as BCs and O-LMs. Modulations or stimulations on HCN or Na_v_ channels will affect neuronal excitability and/or neurotransmission. LTG is known as a sodium channel blocker, which can inhibit IN excitability and, thus, suppress synaptic outputs. However, LTG has been recently reported as an HCN enhancer and was shown to augment neuronal excitability and GABAergic transmission [13]. With these dual conflicting effects on Na_v_ and HCN channels, here, we reported the first investigation on the net effect of LTG on GABAergic inhibition at BC-GC synapses in the DG. As a result, our evidence showed LTG inhibited GABA_A_-mediated transmission by reducing the intrinsic excitability of BCs. In addition, LTG reduced the frequency of sIPSCs, but not mIPSCs (Figure 3), providing evidence that the reduced excitability of presynaptic INs is mediated through Na_v_ channel blockade. Consistently, we showed that LTG greatly decreased AP frequency in fast-spiking BCs in response to prolonged depolarizing current injections (Figure 4B). Therefore, the mechanism is likely through the use-dependent inactivation of Na_v_ channels [8,9,10]. We also examined the effect of LTG on GABAergic inhibition and HCN conductance in the CA1 area. In contrast to the previous results [13], we found that LTG had no effect on both I_h_ current and enhancement of GABAergic transmission. The regulatory mechanism of LTG on GABAergic transmission may be more complicated than we currently understand.

The HCN channel is activated by membrane hyperpolarization and is permeable to both Na^+^ and K^+^ ions with a reversal potential around −30 mV. It tends to offset the membrane hyperpolarization by depolarizing inward current and stabilizes the membrane potential. In addition, the HCN channel remains open and leaky near the resting membrane potential, thus contributing to the membrane conductance [31]. Evidence provided by precious studies indicated that LTG is not only a Na_v_ channel inhibitor, but also a HCN channel enhancer [13,14]. However, our evidence fails to support the role of LTG as an HCN channel enhancer based on the following findings. First, in whole-cell voltage-clamp recordings, the facilitation of I_h_ by LTG was not observed in highly HCN-expressing cells, including O-LM INs and pyramidal cells in the CA1 area (Figure 6H–K); Second, in the whole-cell current-clamp recording acquired in dentate BCs and CA1 O-LM INs, no significant changes were found in the sag ratio and input resistance after application of LTG (Figure 4D–F and Figure 6D–F); Third, in the experiment of spontaneous firing of O-LM INs, we did not find remarkable differences in resting membrane potential following LTG application (data not shown); Fourth, previous studies demonstrated that mIPSCs recorded in GCs mostly originated from synapses close to soma, implying that BCs are the major source of mIPSCs [18]. Moreover, mIPSC frequency recorded in GCs was significantly suppressed by the specific I_h_ channel blocker (ZD7288), suggesting the existence of the I_h_ channel in the axonal terminal of BCs [19]. As an I_h_ enhancer, LTG is expected to depolarize the nerve terminals and enhances the GABA release. However, we did not find any remarkable change in mIPSCs, as well as the uIPSC from BC-GC paired recording following the application of LTG. Therefore, the effect of LTG in this study may be mainly attributed to the blockade of sodium channels.

Imbalanced synaptic excitation and inhibition is an important phenotype of the epileptogenic profile. Thus, modulation of synaptic transmission for promoting global circuit inhibition is thought to be one of the main targets of AEDs. LTG has been noted to facilitate GABAergic inhibition [11,12,13], which appears to be an effective strategy for inhibiting epilepsy and propagation, but the mechanisms remain unclear. Moreover, there are conflicting reports regarding whether GABAergic inhibition is enhanced or suppressed by LTG. In fact, as a Na_v_ channel blocker, earlier studies have indicated that LTG can inhibit both excitatory and inhibitory synaptic events, which occurred spontaneously in cultured neural circuits [32]. In addition, the glutamatergic transmission is much more sensitive to LTG than the GABA system. LTG was reported to inhibit evoked glutamate release with an ED_50_ value of 21 µM, but is less potent in the inhibition of GABA release with an ED_50_ of 44 µM [33,34]. A more recent study revealed that LTG also blocks IPSCs in a dose-dependent manner from amygdala slices [35]. Furthermore, LTG has been observed to preserve the function of GABAergic microcircuits, including feedback inhibition at 25 µM [36].

Our results show that the cIPSC is inhibited significantly by 43.8% in the presence of a high concentration (100 µM) of LTG, whereas it is decreased by 11% and 6% at 30 µM and 10 µM, respectively, which was insignificantly different from the control value. In addition, the cIPSCs were evoked by axonal stimulation in GCL, and these perisomatic GABAergic inputs are derived from three IN subtypes, parvalbumin (PV)-positive fast-spiking INs, such as BCs, axoaxonic cells, and cholecystokinin (CCK)-positive INs. However, CCK INs are present in small populations and have relatively sparse axonal arborization [37]. Therefore, most of the recruited axons via electrical stimulation in GCL come from the PV-positive interneurons, mainly BCs. Moreover, recent studies demonstrate that highly expressed Na^+^ channel exists in BC axon; the gradually increased density of Na^+^ from the soma, proximal axon and then to distal axon contributes to the rapid signaling and reliability of AP propagation [38]. The high density of Na^+^ channels on BC axons may be the reason for the ineffectiveness of LTG at lower concentrations (≤30 μM).

Spontaneous generation of mIPSCs provides a form of tonic or long lasting inhibition [18] and plays a potential role on controlling the excitability of the postsynaptic target cell. In previous reports, LTG has been proposed to inhibit the amplitude of mEPSCs via blocking the postsynaptic AMPA receptor [39]. Our finding indicates that LTG has no effect either on the frequency or amplitude of mIPSCs. Consequently, LTG appears to preferentially depress the tonic excitatory, glutamate-mediated synaptic events, whereas preserving the tonic GABAergic inhibition in the DG.

Consistent with previous reports by pharmacological isolations [39], our evidence also revealed that the reduction of the mean cEPSCs amplitude (acquired by perforant path stimulation in the presence of SR95531) was markedly larger than the cIPSCs at either LTG 30 μM or 100 μM (data not shown), which indicated that LTG preferentially suppressed excitatory neurotransmission in the DG. This could be due to the following reasons. First, fast-spiking BCs largely responsible for GABA release have a different Na channel gating property from principle cells, which contribute to the major glutamate release. Slower inactivation and faster recovery from Na_v_ inactivation in BCs [26] allow LTG to act more easily on PCs and to inhibit much more concurrent glutamatergic transmission; Second, the high density of Na_v_ in GABAergic INs [40] may render GABAergic INs more resistant to LTG than principle cells. However, further investigations are needed to dissect the underlying mechanism of the differential effects of LTG on synaptic excitation and inhibition.

Nevertheless, the alteration of neurotransmitter release is usually associated with the dynamic change of short-term plasticity [41]. The phenomenon of paired pulse or multiple pulse depression has been generally shown on GABAergic synapse [24], as well as our data (Figure S1B,D). Although the multiple pulse ratio (compound IPSC_5_/IPSC_1_) was not affected by LTG (Figure S1D), it is still not certain if LTG preserves GABAergic release at BC-GC synapses, which was characterized by release-independent short-term plasticity [42]. Thus, we further examined the LTG effect on GABAergic release by measuring uIPSC change through BC-GC paired recordings. No significant uIPSC change was found upon LTG application, indicating that LTG has little effect on BC output synaptic release.

Moreover, why was the mean amplitude of sIPSC reduced in CA1 PC (Figure 5A3), but not in dentate GC (Figure 3A3) by the treatment of LTG? We proposed two points of view to discuss this phenomenon. First, sIPSCs consist of both action-potential (AP)-dependent and independent events. For the AP independent events, that is mIPSCs, a lower mean amplitude exists compared to AP-dependent events, as we shown in Figure 5A3 vs. B3 and Figure 3A3 vs. B3. The sIPSC frequency in CA1 (6.43 ± 0.73 Hz) is much higher than that in DG (1.2 ± 0.1 Hz), which indicates that the CA1 interneurons (INs) spontaneously spike at higher frequency. LTG as a use-dependent Na_v_ blocker will preferentially block the AP-dependent (higher-amplitude) events in CA1; thus, the mean amplitude of sIPSC was probably reduced in CA1 PC, but not in dentate GC by LTG. Second, in the view of the local circuit, the CA1 PCs receive numerous and diverse GABAergic inputs from distinct local INs, including soma-targeting INs (S-INs) and dendrite-targeting INs (D-INs), which usually show burst firing at the gamma frequency band [43,44]. Synapses of S-IN usually display a high release probability of first release and depressing (paired pulse ratio (PPR) or multiple pulse ratio <1) short-term dynamics; whereas synapses of D-IN exhibit a relatively low release probability of first release and facilitating (PPR > 1) short-term dynamics [23,42,45]. Therefore, in burst spiking of the dendrite-targeting INs, LTG may selectively inhibit the synaptic responses (events with a larger amplitude due to multiple pulse facilitation) elicited by later action potentials. Therefore, application of LTG reduced the mean amplitude of sIPSC in CA1 PCs (Figure 5A3). Nevertheless, dentate GCs receive major GABAergic inputs from soma-targeting INs [18]. The selective inhibition of LTG may be minimized in this local circuit, and this is also consistent with the results of the unchanged mean amplitude of sIPSC in GCs shown in Figure 3A3.

In conclusion, we revisited the effects of LTG on GABAergic inhibition, as well as the intrinsic properties of upstream interneurons under the normal condition of rodent hippocampus. The results show that LTG likely behaves as a use-dependent Na^+^ channel blocker, while it has no significant action on I_h_ potentiation. Even though our finding is contrary to previous reports, we still look forward to a reliable compound with specific targeting of the HCN channel as a potent enhancer, which will have therapeutic potential for more neuropsychological and cardiovascular disorders.

## 4. Materials and Methods

### 4.1. Acute Brain Slice Preparation

Male Sprague-Dawley rats (postnatal 16–21 days) were decapitated with isoflurane anesthesia in agreement with the national and institutional guidelines, and all procedures were approved by the Animal Care and Use Committee of National Yang-Ming University. After rapid removal of the brain, transverse slices were sectioned in ice-cold cutting buffer containing (in mM) 87 NaCl, 25 NaHCO_3_, 1.25 NaH_2_PO_4_, 2.5 KCl, 10 glucose, 75 sucrose, 0.5 CaCl_2_ and 7 MgCl_2_ using a microslicer (DTK-1000, Dosaka, Kyoto, Japan), recovered (30 min, 34 °C) in the cutting buffer (oxygenated with 95% O_2_/5% CO_2_), then stored at room temperature (22–24 °C). During the experiments, slices were transferred to a submersion recording chamber and perfused with oxygenated artificial cerebrospinal fluid (ACSF) containing (in mM): 125 NaCl, 25 NaHCO_3_, 1.25 NaH_2_PO_4_, 2.5 KCl, 25 glucose, 2 CaCl_2_ and 1 MgCl_2_.

### 4.2. Electrophysiology

Patch pipettes for recordings were pulled from borosilicate glass tubing (outer diameter 1.5 mm, inner diameter 0.86 mm; Harvard apparatus, Holliston, MA, USA) and heat-polished before being used. The pipette resistance normally ranged from 3–5 MΩ. Experiments were performed under visual control using an infrared differential interference contrast (IR-DIC) microscope (BX51WI, Olympus, Tokyo, Japan). GCs of low input resistance (R_in_ < 600 MΩ) were chosen for recordings. Whole-cell patch recordings were made as described previously [27] using Multiclamp 700B or Axopatch 200B amplifiers (Molecular Devices, Union City, CA, USA).

In voltage clamp experiments, compound IPSCs (cIPSCs) were evoked by a glass stimulation pipette (~20 µm tip diameter filled with ACSF) placed in the GC layer (GCL) in the presence of 2 mM kynurenic acid (KA) (an ionotropic glutamate receptor blocker). Pulses were delivered every 10–15 s by a stimulus isolator (Isoflex, A.M.P.I.). In addition, spontaneous synaptic responses (ex. sIPSCs) were recorded in a similar condition, whereas the miniature responses (ex. mIPSCs) were measured in the presence of TTX (1 μM). The holding potential was set to −70 mV, and data were discarded if the changes of the series resistance changes exceeded 20%. Signals were low-pass filtered at 2 kHz (four-pole Bessel) and sampled at 10 kHz using Digidata 1440 (Molecular Devices); data acquisition and pulse generation were done using pClamp 10.2 (Molecular Devices).

In current-clamp experiments, the resting membrane potential was set near −70 mV by injecting negative holding currents. We measured the intrinsic excitability of INs by delivering depolarizing current steps in the presence of KA (2 mM) and SR95531 (1 μM). For the experiment of the stimulation antidromic AP generation, a unipolar electrode was placed in the GCL at a distance of 100–300 μm from the soma of the recorded BC. Paired recordings from synaptically-connected BCs and GCs in the DG were made as described previously [22,23]. Presynaptic INs were held near −70 mV in current clamp. Multiple short (1 ms) current pulses were delivered to evoke clustered APs at 10-second intervals. Postsynaptic cells were held at −80 mV in voltage clamp. Whole-cell patch-clamp recordings were made using a Multiclamp 700B amplifier (Molecular Devices). Pipette capacitances of both electrodes were carefully compensated (by >95%), and series resistance (Rs) was compensated using the automatic bridge balance (the readouts after compensation were 9–28 MΩ).

### 4.3. Morphological Identification

Neurons were filled with biocytin (2 mg/mL) during whole-cell recordings and subsequently fixed overnight with 4% paraformaldehyde in phosphate-buffered solution (PB; 0.1 M, pH 7.3). After washing with PB, slices were incubated with fluorescein isothiocyanate-conjugated avidin-d (2 µL/mL; Invitrogen, Eugene, OR, USA) in PB and 0.3% Triton X-100 overnight at 4 °C. After washing, slices were embedded in mounting medium Vectashield^®^ (Vector Laboratories, Burlingame, CA, USA). Labeled cells were imaged by a two-photon microscope as described before [25]. The two-dimensional morphology of the cells was reconstructed from a stack of 100 images (voxel size, 0.378–1.514 μm in the *x–y* plane; 0.4–0.99 μm along the *z*-axis) using ImageJ (v.1.42q) or Neuromantic 1.6.3 software.

### 4.4. Solutions and Drugs

The intracellular solution used in GCs electrophysiological recordings for IPSCs contained (in mM): 140 KCl, 10 EGTA, 2 MgCl_2_, 2 Na_2_ATP and 10 HEPES; pH adjusted to 7.3 with KOH (Aponte et al., 2006). The intracellular solution for CA1 PCs consisted of (in mM): 129 CsCl, 10 HEPES, 10 EGTA, 2 MgCl_2_, 0.3 NaATP and 4 MgATP (pH adjusted to 7.3; 280–290 mOsm) (Peng et al., 2010). The intracellular solution for IN patch recordings contained (in mM): 135 K-gluconate, 20 KCl, 0.1 EGTA, 2 MgCl_2_, 4 Na_2_ATP, 10 HEPES and 0.3 Na_3_GTP; pH adjusted to 7.3 with HCl. Gabazine (SR95531) was purchased from Tocris Bioscience (Park Ellisville, MO, USA); ZD7288, LTG and all other chemicals were purchased from Sigma (St. Louis, MO, USA), except where noted.

### 4.5. Data Analysis and Statistics

Data were analyzed using Clampfit 10.2 and GraphPad Prism 5.0. sIPSCs and mIPSCs were detected using Mini-Analysis (Synaptosoft). All values were given as the mean ± SEM. Error bars indicated SEM. The significance test for comparison between pre- and post-drug used was the non-parametric paired difference test (Wilcoxon signed-rank test) and the Kolmogorov–Smirnov two-sample test (K-S test). Two-way ANOVA was applied to compare AP frequency or I_h_ between control and LTG groups over the repeated measurement under the incremental current step or voltage step protocol, respectively. A *p*-value less than 0.05 was considered statistically significant. All statistical analyses were conducted with SPSS 18.0 (SPSS Inc., Chicago, IL, USA). For the data of cIPSCs, concentration-response curves were fitted with the Hill equation, $f\left(c\right)=\frac{A}{\left[1+{\left(\frac{{\mathrm{EC}}_{50}}{c}\right)}^{n}\right]},$ where A is the constant for the maximal effect, *c* denotes the concentration, EC_50_ represents the half-maximal effective concentration and *n* means the Hill coefficient.

## Supplementary Materials

Supplementary materials can be found at http://www.mdpi.com/1422-0067/17/7/1191/s1.

## Abbreviations

LTG	lamotrigine
IN	interneuron
HCN	hyperpolarization-activated cyclic nucleotide
BC	basket cell
GC	granule cell
IPSC	inhibitory postsynaptic current
PC	pyramidal cell
I_h_	hyperpolarization-activated current
AED	antiepileptic drug
AP	action potential
ACSF	artificial cerebrospinal fluid
KA	kynurenic acid
DG	dentate gyrus
