# Supplementary material for: Revisiting the Lamotrigine-Mediated Effect on Hippocampal GABAergic Transmission
Source: Int J Mol Sci. 2016 Jul 22;17(7):1191. doi: 10.3390/ijms17071191 (PMC4964560; doi:10.3390/ijms17071191)
Supplement: Supplementary file 1 [file ijms-17-01191-s001.pdf]

Supplementary Materials: Revisiting Lamotrigine-Mediated Effect on Hippocampal GABAergic Transmission

Yu-Yin Huang, Yu-Chao Liu, Cheng-Ta Lee, Yen-Chu Lin, Mong-Lien Wang, Yi-Ping Yang, Kaung-Yi Chang and Shih-Hwa Chiou

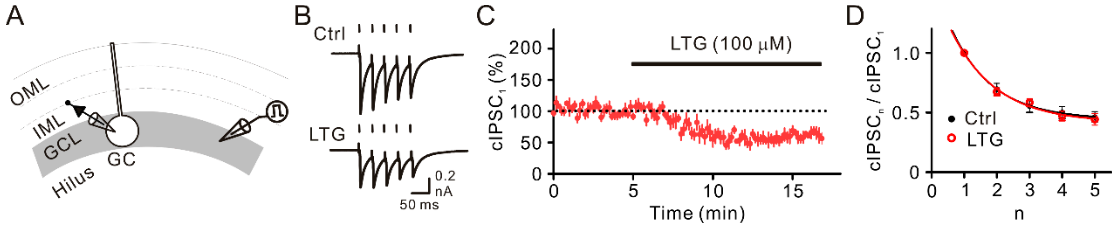

Figure S1. LTG inhibited compound IPSCs without affecting multiple pulse ratio at BC-GC synapse. (**A**) Schematic diagram showing extracellular stimulation of IN axons in the GCL and GC recording; (**B**) Compound IPSCs were evoked by stimulation protocol (25 Hz burst of 5 current pulses every 10 s) and the corresponding cIPSC traces (average of 25 sweeps) before and after treatment of LTG (100 μM). Note that LTG did not alter short-term synaptic plasticity. Vertical ticks represent the stimulation pulses; (**C**) Plot of their corresponding normalized cIPSC1 amplitude in control and after bath perfusion of LTG (100 μM) from 7 experiments. Horizontal bar represents time of LTG application. Symbols indicate mean; error bars indicate SEM; (**D**) Mean ratio of cIPSCn/cIPSC1 plotted against the number within the train (*n*).
